# Occipital shuntalgia: Rethinking post-shunt occipital headache etiology and care

**DOI:** 10.1007/s00701-026-06797-4

**Published:** 2026-02-18

**Authors:** Shachar Zion Shemesh, Noa Rennert, Zeev Feldman, Paz Kelmer, Itay Goor-Aryeh, Oded Jacobi, Gabriel Lichtenstein, Yotam Hadari, Zvi R. Cohen, Lior Ungar

**Affiliations:** 1 Department of Neurosurgery, Sheba Medical Center, Ramat Gan, Israel; 2 Sackler Faculty of Medicine, Tel Aviv University, Tel Aviv, Israel; 3 Pain Center, Sheba Medical Center, Ramat Gan, Israel

**Keywords:** Shunt, Pain, Neuralgia, Headache

## Abstract

**Objective:**

Headache after ventriculoperitoneal (VP) shunting is classically attributed to CSF over-drainage or shunt malfunction. We hypothesized that a subset of adults instead experience an occipital neuralgia-like pain syndrome (“Occipital Neuralgia”) from shunt hardware irritating the occipital nerves, and that these cases respond better to nerve-targeted treatments than to shunt revisions.

**Methods:**

We retrospectively reviewed 2,223 adults who underwent first-time VP shunt placement between 2000 and 2025 at a tertiary center. Patients with post-shunt headache were identified and classified as Occipital Neuralgia if they had occipital-predominant, lancinating pain with focal tenderness over the valve or tract, short-term relief from diagnostic occipital nerve block, and no evidence of over-drainage, malfunction, or infection on work-up. Clinical features, management, and outcomes (nerve blocks, neuromodulation, shunt adjustments or revisions) were compared between Occipital Neuralgia and other post-shunt headaches.

**Results:**

Among 2,223 adults who underwent VP shunt placement, 32 patients (1.44%) developed new, persistent post-shunt headaches not attributable to shunt malfunction, over-drainage, or infection. Of these, 24 patients (1.08% of the total cohort; 75% of chronic post-shunt headaches) met criteria for Occipital Neuralgia. These patients typically presented with occipital-predominant, lancinating pain, focal scalp tenderness over the shunt valve or tract, and absence of orthostatic features. Neuroimaging demonstrated normal or slit ventricles without signs of intracranial hypotension or other structural intracranial pathology. Most patients experienced substantial symptomatic improvement following nerve-targeted therapies, whereas shunt-directed interventions provided limited benefit once pressure-related causes were excluded.

**Conclusions:**

Post-shunt occipital neuralgia is a recognizable, under-appreciated cause of headache after VP shunting. Early recognition of a focal occipital neuropathic phenotype and nerve-targeted therapy can yield meaningful relief and help avoid unwarranted shunt revisions. Prospective validation of diagnostic criteria and management pathways is needed.

## Introduction

Headache is a well-recognized sequela of CSF shunting for hydrocephalus. Traditional paradigms emphasize pressure-related mechanisms, such as over-drainage headaches in the *slit ventricle syndrome* spectrum, which often present with orthostatic exacerbation and characteristic neuroimaging findings (collapsed ventricles, pachymeningeal enhancement, subdural fluid collections). In practice, work-up of a shunt-treated patient’s headache typically prioritizes ruling out shunt malfunction or over-drainage, since shunt failures and siphoning can cause severe positional headaches requiring intervention. However, headaches in shunt-dependent patients are not uniformly due to shunt malfunction or low pressure. Retrospective data indicate that a substantial proportion of shunted patients experience chronic headaches despite a functioning shunt [1, 2, 4, 5]. Misdiagnosis in these cases can lead to costly or unnecessary shunt revisions. Indeed, even when shunts are working properly, patients may suffer headaches from other etiologies.

Clinicians have noted a distinctive subset of post-shunt headaches that do *not* fit the typical over-drainage profile. These patients report predominantly occipital, often unilateral pain described as lancinating or stabbing, with focal scalp tenderness or Tinel-like dysesthesias over the shunt valve or subcutaneous tubing tract, and radiation in the distribution of the greater and/or lesser occipital nerves (C2-C3 dermatomes). This phenotype closely aligns with occipital neuralgia, a cranial neuropathic pain syndrome defined by paroxysmal shooting pain in the posterior scalp in the territory of the occipital nerves, accompanied by point tenderness over the nerve and temporary relief with local anesthetic blockade. Occipital neuralgia is most often idiopathic or associated with trauma but has also been reported as an iatrogenic pain syndrome after cranial surgeries (craniotomy, Chiari decompression) or cervical instrumentation. In the context of VP shunts, an occipital neuropathic pain mechanism is biologically plausible: the rigid shunt hardware (valve or catheter) traversing the suboccipital scalp may irritate or compress the occipital nerve fibers, analogous to an entrapment neuropathy. Pediatric case series have provided proof-of-concept: occipital nerve blocks relieved shunt-related occipital headaches in children who had often undergone multiple shunt revisions. In one report of 7 pediatric patients with VP shunts and chronic occipital headaches, all had improvement after greater occipital nerve block, including a subset with long-lasting relief or successful subsequent nerve ablation, and without shunt revision. These observations suggest that a “hardware-nerve” interaction can cause clinically significant headache independent of intracranial pressure status [7–10].

Adult data on this phenomenon remain sparse. While chronic headaches are known to occur in adults with long-standing shunts (for example, more than one-third of iNPH patients report recurrent headaches post-shunting), the specific contribution of occipital nerve irritation has not been well characterized in the adult population. We postulated that *Occipital Neuralgia* represents a distinct clinicopathological entity in adults: an occipital neuralgia-like post-shunt headache due to peripheral nerve irritation by the shunt hardware. We further hypothesized that [19] Occipital Neuralgia may account for a significant fraction of persistent post-shunt headaches in adults, [6, 20–23] these headaches would preferentially respond to nerve-targeted treatments (nerve blocks, ablations, or neuromodulation) once intracranial pressure abnormalities are excluded, and [11–18] anatomical factors such as shunt hardware trajectory or occipital nerve proximity could influence risk. To test these hypotheses, we conducted a 25-year retrospective review of adult VP shunt cases, identifying those with post-shunt headaches and characterizing a subset meeting predefined Occipital Neuralgia criteria. We compared clinical features and outcomes between Occipital Neuralgia and other post-shunt headaches, and we report on the management strategies utilized. Our goal is to provide the first comprehensive description of Occipital Neuralgia in adults, examine its relationship to historical pediatric observations, and outline a management framework that may improve care and avoid unnecessary surgeries.

## Methods

### Study design and setting

We performed a retrospective cohort study at a single academic neurosurgical center with an integrated chronic pain service. The study was approved by the institutional review board (IRB), which granted a waiver of informed consent due to the retrospective design and exclusive use of de-identified patient data.

### Patient population

All adults (age ≥ 18 years) who underwent first-time VP shunt placement at our institution between January 1, 2000 and December, 2025 were eligible for analysis. Patients were identified from operative databases and ICD procedural codes for CSF shunt insertion. This yielded a cohort of 2,223 adult patients with new VP shunts. We screened neurosurgery and pain clinic records for documentation of post-shunt headache episodes. Initially, all patients with new or worsening headache after shunt placement were identified, including those with pressure-related etiologies (e.g., over- or under-drainage, shunt malfunction, or infection).

To construct a clinically coherent cohort for this study, we then excluded patients whose headaches were clearly attributable to pressure abnormalities and resolved after shunt-directed management (valve adjustment, addition of anti-siphon devices, shunt revision, or treatment of shunt infection).

The remaining 32 adults (1.44% of the shunt cohort) had persistent, daily post-shunt headaches that were *not* explained by shunt over- or under-drainage, malfunction, or infection and did not resolve despite appropriate shunt optimization. These 32 patients comprised the analytic “chronic non-pressure post-shunt headache” cohort. Within this group, 24 met prespecified criteria for *Occipital Neuralgia*, and 8 were classified as “other post-shunt headache” for comparison.

### Rationale for operational criteria

Our operational criteria for Occipital Neuralgia differ from the International Classification of Headache Disorders, 3rd edition (ICHD-3) criteria for occipital neuralgia for several reasons specific to the retrospective design and clinical context of this study. First, ICHD-3 criteria require systematic documentation of specific examination findings (tenderness to palpation over the occipital nerves, triggering of pain by pressure on the emergence site) that were not consistently recorded during routine clinical encounters. Our criteria were designed to be applicable to retrospective chart review where documentation varies across providers and visits. Second, the ICHD-3 requirement to exclude alternative headache diagnoses is difficult to apply retrospectively without structured headache questionnaires. Third, the post-shunt setting necessitated an additional criterion not present in ICHD-3: explicit exclusion of shunt-related pressure abnormalities (malfunction, over-drainage, infection), which is critical in this population but irrelevant to primary occipital neuralgia. Our approach prioritized positive identification of neuralgiform occipital pain in proximity to shunt hardware, combined with response to nerve-targeted therapy, as pragmatic criteria that could be reliably extracted from existing medical records.

### Operational definition of occipital neuralgia

The operational criteria used in this study were intentionally designed to capture a clinically meaningful, shunt-associated occipital neuropathic pain syndrome in a retrospective setting, rather than to strictly replicate the International Classification of Headache Disorders, 3rd edition (ICHD-3) definition of occipital neuralgia. While ICHD-3 criteria are optimized for prospective diagnostic classification, several required elements (such as detailed paroxysmal pain characterization or systematic documentation of trigger maneuvers) are often incompletely recorded in routine neurosurgical practice. Therefore, we adopted pragmatic criteria emphasizing anatomical pain distribution, focal examination findings, exclusion of pressure-related shunt pathology, and when available response to occipital nerve block, which together were felt to best reflect the clinically relevant phenotype encountered in shunted patients.

Occipital Neuralgia was defined by the presence of all of the following three criteria:

1. Occipital nerve distribution pain with neuralgiform quality: Predominantly occipital location of pain (overlapping the mapped course of the greater and/or lesser occipital nerves), with paroxysmal lancinating or stabbing character. On examination, there is focal tenderness or allodynia over the occipital nerve course and/or the area of the shunt valve or catheter tract in the occipital region.
2. Supportive response to occipital nerve block: When an occipital nerve block (ONB) was performed, it provided transient but clear relief of the headache, consistent with a diagnosis of occipital neuralgia (this criterion was waived for a few patients in whom a block was not performed, as long as other criteria were met).
3. Exclusion of pressure-related causes: Clinical and radiological evaluation did not reveal any evidence of shunt malfunction, over-drainage, or shunt-related infection at the time of headache onset. All patients had neuroimaging (CT or MRI) reviewed for ventricular size and signs of intracranial hypotension (e.g. diffuse pachymeningeal enhancement, venous sinus engorgement, subdural hygromas or hematomas, cerebellar tonsillar descent). Shunt patency was confirmed by flow study or reservoir tap when indicated. Standard evaluations ruled out shunt infection (no fever, incision inflammation, or CSF pleocytosis if tapped). Patients whose headaches resolved after treatment of a clear shunt malfunction or over-drainage were not considered to have Occipital Neuralgia.

Patients who fulfilled all the above criteria were classified as Occipital Neuralgia. Those with post-shunt headache who did not meet criteria (for example, headaches with migrainous or tension-type features, or attributable to another cause such as intracranial hypertension or recurrent hydrocephalus) were classified as “other post-shunt headache” for this analysis.

Standard VP shunt implantation techniques were used throughout the study period. In adult patients, ventricular catheter placement was performed predominantly via a right frontal burr hole, with occipital entry reserved for select cases based on surgeon preference or anatomical considerations. The ventricular catheter was connected to a subcutaneous valve-reservoir system, which was typically positioned in the parietal or retroauricular region, followed by distal catheter tunneling to the peritoneal cavity. Shunt systems included both programmable and fixed-pressure valves, with or without adjunct anti-siphon or gravitational devices, according to the treating neurosurgeon’s preference and the underlying hydrocephalus etiology. Valve types and initial pressure settings were documented and are summarized in Table 1.

**Table 1:** Baseline characteristics of shunted adults with no headache versus those with post-shunt headaches, including the Occipital Neuralgia subgroup

Characteristics	No unexplained* post-shunt headache (*n* = 2,191)	Any unexplained Post-Shunt Headache (*n* = 32)	Occipital Neuralgia (*n* = 24)
Age at shunt implantation, years	50.05 ± 14	49.87 ± 13	49.95 ± 7
Male sex	1,299 (59.3%)	14 (43.8%)	13 (54.1%)
Hydrocephalus etiology:			
- Normal pressure hydrocephalus	1052	20	11
- Aqueductal stenosis	174	0	1
- Subarachnoid hemorrhage	402	8	5
- Tumor-related	450	3	5
- Idiopathic intracranial hypertension	20	0	0
- Other	93	1	3

* “Unexplained” indicates that no recognized shunt-related etiology was identified after standard evaluation (e.g., over/under-drainage, malfunction/obstruction, infection, slit ventricle syndrome)

Values are presented as mean ± standard deviation or count (%)

### Data collection

We abstracted clinical data from the electronic medical record, including patient demographics (age, sex) and hydrocephalus etiology (categorized as: normal pressure hydrocephalus [NPH]; aqueductal stenosis; subarachnoid hemorrhage [aneurysmal or traumatic]; brain tumor-related hydrocephalus; idiopathic intracranial hypertension [IIH]; and other causes such as congenital or post-infectious hydrocephalus). Shunt hardware characteristics were recorded for all patients: cranial entry site (frontal vs occipital, and laterality), valve type (programmable vs fixed-pressure; valve model and opening pressure setting), use of anti-siphon or gravitational accessory devices at initial placement, and the position of the valve/reservoir relative to the occipital nerve corridor (i.e. whether the hardware lay in the suboccipital region near the superior nuchal line).

For patients with post-shunt headaches, we collected detailed headache phenotype information from clinic notes: time from shunt surgery to headache onset, pain location and quality (neuralgiform descriptors such as “stabbing” or “electric” vs other descriptors), presence or absence of orthostatic pattern (worsening when upright), radiation to the frontal or orbital regions (which can occur via trigeminocervical convergence [16]), occipital nerve territory sensory changes, scalp tenderness or Tinel’s sign over the tract, and any other autonomic or migrainous features. Pre-shunt headache history (if any) was noted. Headache-related disability was assessed using standard scales when available: Headache characterization focused on diagnostic criteria for occipital neuralgia (ICHD-3-compatible clinical features and exam findings). Pain intensity was abstracted using a 0–10 Numerical Rating Scale (NRS) when documented in the record.

Imaging and shunt evaluations were reviewed for each headache case. As described, all had head CT or MRI assessing ventricular size (categorized as slit, normal, or enlarged relative to baseline imaging) and signs of intracranial hypotension or elevated pressure. Shunt series X-rays were reviewed for catheter continuity. When headache evaluation included an in-office shunt tap or adjustment, we recorded opening pressure and any diagnostic findings (e.g. “slow refill” of the reservoir on manual compression, which can signify over-drainage).

Interventions for headache management were documented. We categorized treatments as “nerve-targeted” versus “shunt-targeted.” Nerve-targeted treatments included occipital nerve blocks (local anesthetic with or without steroid, performed by the pain specialist), radiofrequency ablation of the occipital nerves (pulsed RF at 42 °C for 120 s, in our practice), and occipital nerve stimulation (ONS) in refractory cases. Shunt-targeted treatments included adjustments of the programmable valve setting, addition of anti-siphon or gravitational units to the shunt system, or surgical revision/relocation of the shunt catheter or valve. For each intervention, we noted the outcome in terms of headache relief (categorized as ≥ 50% pain reduction, < 50% improvement, or no change/worsening) and any complications. Duration of response to nerve blocks (in weeks) was recorded from clinic follow-up. Finally, overall follow-up time from shunt insertion and from headache diagnosis was recorded. We noted any recurrence of headache after initial successful therapy, to analyze durability.

No structured headache assessment template was used during routine clinical care. Clinical features were prospectively documented as part of standard neurosurgical and pain clinic encounters and retrospectively abstracted for the purposes of this study. Variables related to pain location, focal tenderness, imaging findings, and treatment response were generally well documented, whereas detailed temporal headache characteristics were inconsistently recorded. Missing data were handled by complete-case descriptive reporting without imputation.

### Outcomes and statistical analysis

The primary outcome measures were: (1) the proportion of Occipital Neuralgia cases among all VP-shunted adults and among those with any post-shunt headache; and (2) the comparative response rate of Occipital Neuralgia to nerve-targeted treatments versus shunt-directed interventions, defined as the percentage of each yielding ≥ 50% pain relief.

Descriptive statistics are presented as mean ± standard deviation (SD) or median for continuous variables, and counts (percentage) for categorical variables. Group comparisons (no-headache vs headache, and Neuralgia vs other headache) utilized t-tests or Wilcoxon rank-sum tests for continuous variables and chi-square or Fisher’s exact tests for proportions, as appropriate. Time-to-event (headache recurrence) was analyzed using Kaplan–Meier curves; a Cox proportional hazards model was considered if sample size allowed. Statistical significance was defined at α = 0.05 (two-tailed). All analyses were performed using SPSS and PyCharm.

## Results

### Cohort overview

Over the 25-year period, 2,223 adults underwent first-time VP shunt placement at our institution. Among these, numerous patients experienced post-shunt headaches related to over- or under-drainage, malfunction, or infection, which typically improved after shunt optimization and were not analyzed further.

**Fig. 1:**
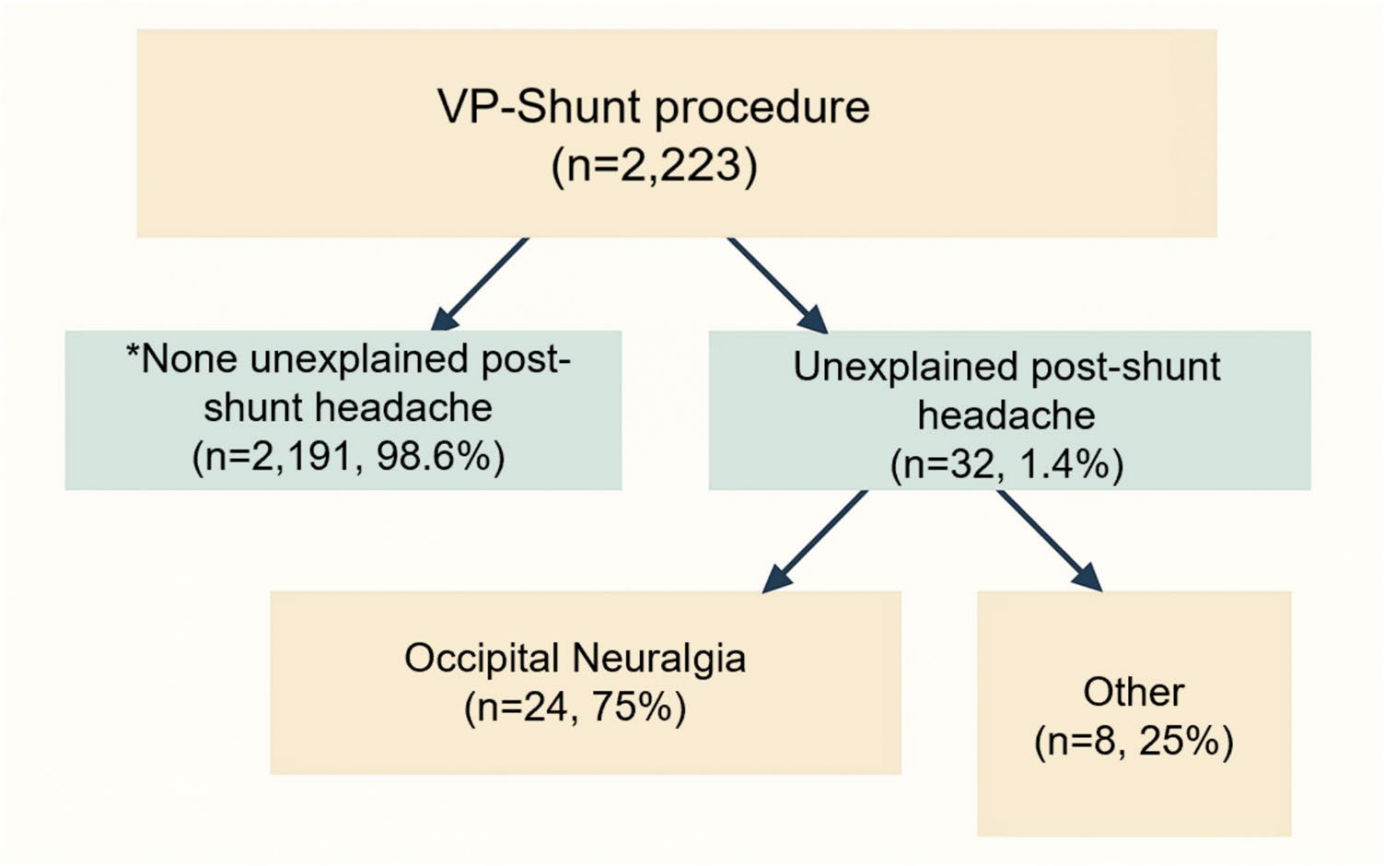
Flow diagram of the study cohort. Among 2,223 VP shunt procedures, 2,191 patients (98.6%) had no unexplained post-shunt headache*, while 32 (1.4%) had unexplained post-shunt headache*. Within the unexplained headache cohort, 24 patients (75%) met criteria for Occipital Neuralgia, and 8 (25%) were classified as other. * “Unexplained” indicates that no recognized shunt-related etiology was identified after standard evaluation (e.g., over/under-drainage, malfunction/obstruction, infection, slit ventricle syndrome)

Thirty-two patients (1.44%) fulfilled our definition of chronic, non-pressure post-shunt headache that persisted despite a functioning shunt system and appropriate shunt-directed management. These 32 patients constitute the cohort described in this report. Figure 1 illustrates the cohort flow. Of the 32 post-shunt headache patients, 24 cases (1.08% of the total shunt cohort) fulfilled criteria for Occipital Neuralgia, representing 75% of all post-shunt headaches. The remaining 8 patients (0.36% of shunt cohort; 25% of post-shunt headaches) had other headache types (hereafter “Other Headache” group). Importantly, in all 32 cases the headaches represented new, chronic symptoms that emerged only after shunt surgery despite a functioning shunt system, consistent with a post-operative complication rather than shunt failure or over-drainage.

All 32 headache patients initially presented with concerns for shunt malfunction or over-drainage, but true shunt failures were ruled out in these cases. In the Occipital Neuralgia subset, thorough evaluation confirmed shunt functionality and no intracranial hypotension, redirecting attention to a neuropathic pain mechanism.

### Patient characteristics

Baseline characteristics of patients with and without post-shunt headache are summarized in Table 1. Patients who developed post-shunt headaches tended to be slightly younger than those who did not (mean age ~ 49.9 vs 50.1 years), but this difference was not significant (*p* = 0.15). The headache cohort had a higher proportion of females (56.3% vs 40.7% female in no-headache group. though males still accounted for 43.8% of the headache group. Among the Occipital Neuralgia cases specifically, 55.9% were female. Hydrocephalus etiologies were diverse; the most common causes in both headache and no-headache groups were normal-pressure hydrocephalus and tumor-related or post-subarachnoid hemorrhage hydrocephalus, reflecting the overall adult hydrocephalus population. We observed no clear over-representation of any etiology in the headache cohort, although the small numbers of limit conclusions (etiology breakdown by group is detailed in Table 1). Notably, 2 of the 24 Neuralgia patients (8%) had a prior history of headaches (both had migraine-type headaches unrelated to the occipital area), whereas the other 22 (92%) had never suffered chronic headaches before shunting. In the Other Headache group (*n* = 8), 2 patients had a history of headaches (e.g. migraine or tension-type) prior to shunt placement.

The shunt hardware profiles were similar between groups in terms of initial valve type and settings One noteworthy difference was the cranial insertion site of the shunt: only 3 of the 32 headache patients (9.4%) had an occipital burr hole for ventricular catheter placement, whereas the vast majority (90.6%) had frontal entry (almost all on the right side). By comparison, in the entire cohort of 2,223, approximately 5% had occipital vs 95% frontal entry (surgeon preference at our center has been a right frontal approach for adult shunts in most cases). Thus, having an occipital entry site was not a prerequisite for developing Occipital Neuralgia; in fact, 21 of 24 Neuralgia patients (87.5%) had frontal shunt entry.

### Shunt hardware and occipital anatomy

Although shunt entry site did not correlate with headache occurrence, we examined the spatial relationship of the shunt hardware to the occipital nerve pathways. Among the 24 Occipital Neuralgia cases, the implanted valve-reservoir was in the retrosauricular (mastoid) region in 2 cases and more anterior (parietal region) in 22 cases. In 83% of Neuralgia patients, the distal catheter’s subcutaneous course ran across the occipital scalp (near the midline or upper cervical region) where the greater occipital nerve ascends. Cadaveric studies have shown that the greater occipital nerve typically crosses the superior nuchal line within 5–30 mm of the midline; thus, shunt hardware placed in that corridor can plausibly irritate the nerve. In all 3 Neuralgia patients with occipital burr holes, the valve was also in the upper occipital region, directly overlying the expected route of the occipital nerves. In contrast, none of the 8 “Other Headache” patients had hardware in the occipital nerve vicinity (all had frontal entry and the valve on the side of the head, away from midline). This anatomical observation supports the hypothesis that hardware proximity to the occipital nerve increases the risk of Occipital Neuralgia, regardless of whether the ventricular entry point is frontal or occipital.

Valve type (programmable vs fixed pressure) and initial opening pressure settings were similar across groups and did not show any clear association with the development of post-shunt headache or Occipital Neuralgia in this retrospective series.

### Headache phenotype

The 24 Occipital Neuralgia patients presented with occipital neuralgia-like pain. The headache began on average [3] weeks after shunt surgery (range [4–16] weeks). Only a minority (6/24, 25%) reported a “classic” neuralgic pattern of sudden, intermittent stabs or “electric shocks” in the occipital region, sometimes superimposed on a dull ache. The majority (18/24, 75%) described a more continuous, non-classic occipital pain with superimposed sharp exacerbations or pressure-like discomfort. This atypical presentation makes the syndrome easier to overlook in routine practice.

### Imaging and shunt evaluations

All Occipital Neuralgia patients had brain imaging around the time of headache presentation. Ventricular size on MRI/CT was normal or slightly small in all 24; none had ventriculomegaly to suggest shunt failure. None showed the diffuse dural enhancement or venous engorgement typical of intracranial hypotension. No other concomitant intracranial pathologies, including tumors, vascular lesions, or other structural abnormalities, were identified on imaging. None of Neuralgia patients had small extra-axial fluid collections, these were stable and asymptomatic, not exerting mass effect.

Shunt series X-rays did not demonstrate any disconnections or fractures in this group. Two patients underwent exploratory shunt revision early in their headache course (at outside hospitals) because of symptom persistence; both were found to have patent shunts intraoperatively, and their headaches continued post-revision until occipital nerve treatment was pursued. By study criteria, these two would still be categorized as Occipital Neuralgia (the initial misinterpretation of their headache as a shunt failure led to an unnecessary revision). We emphasize that none of the Neuralgia patients had true infective or mechanical complications—their CSF studies (when done) were unremarkable, and symptoms did not improve with antibiotic or shunt revision, respectively. The Other Headache patients had variable investigations: at least half had radiological evidence of over-drainage or intermittent ventricular enlargement leading to eventual shunt revision or adjustment, whereas others were diagnosed with primary headache disorders.

### Treatment response and follow-up

All 24 patients diagnosed with Occipital Neuralgia underwent image-guided (fluoroscopy or ultrasound) diagnostic greater occipital nerve block to avoid shunt injury. Four were lost to follow-up. Among those with follow-up, complete pain resolution occurred after 1–3 injections in several cases (two after a single injection, one after two injections, and two after three injections). Additional patients experienced ≥ 50% improvement with scheduled repeat blocks every 2–3 month, or with a combination of neuropathic medications (gabapentinoids or tricyclics) and repeat blocks; a further subgroup improved ≥ 50% with medications alone. Three patients remained refractory despite blocks, medications, and medical cannabis; two of them underwent shunt revision with hardware repositioning, and one of these subsequently experienced marked improvements. No serious device-related complications were observed with nerve-targeted therapies.

In contrast, management in the Other Headache group (*n* = 8) varied according to etiology: several patients with orthostatic features underwent valve adjustment or shunt revision for suspected over-drainage, while others were treated for primary headache disorders.

## Discussion

Our experience suggests that Occipital Neuralgia represents a distinct and clinically important mechanism of post-shunt headache in adults. Although overall incidence of post-shunt headache was low, most of such patients in our series presented with a neuralgiform occipital syndrome rather than pressure-related phenomena. Notably, most patients in our series did not present with the “classic” paroxysmal electric-shock phenotype; instead, they described more continuous occipital pain with superimposed stabbing episodes, a pattern that is easily misattributed to non-neuropathic causes and may contribute to under-recognition of this complication.

This reinforces the concept, first hinted at in pediatric cohorts, that focal irritation of occipital nerves by shunt hardware can produce disabling pain despite a normally functioning system. Recognizing this syndrome reframes the approach to diagnosis and management, redirecting attention from repeated imaging and shunt revisions toward peripheral nerve-focused strategies.

The phenotype is highly characteristic: lancinating occipital pain, often unilateral, with focal tenderness or Tinel-like dysesthesias over the valve pocket or subcutaneous tract. These features are fundamentally different from the positional headaches of over-drainage or the diffuse pressure of malfunction, and their presence should prompt suspicion of a neuropathic origin. Our findings also underscore that the cranial entry point is not the relevant factor; most cases followed frontal shunt placement, with symptoms arising from the distal subcutaneous course crossing the mastoid and upper neck, precisely where occipital nerves run. Anatomical variability in nerve course likely explains why only a minority of shunted patients develop Neuralgia, but in those affected, examination along the tract can reveal diagnostic tenderness that strongly predicts therapeutic response.

Therapeutic outcomes confirm this mechanism. Occipital nerve blocks provided immediate and reproducible relief in most patients, both confirming diagnosis and offering a low-risk initial treatment. Pulsed radiofrequency extended the benefit for months in two-thirds, and occipital nerve stimulation, though used rarely, yielded durable control in the most refractory cases. By contrast, shunt-directed adjustments, antisiphon additions, and even revisions produced negligible benefit once pressure complications were excluded. This divergence highlights the risk of misinterpretation: treating presumed siphonopathy when the problem is neuralgia exposes patients to unnecessary procedures with little chance of improvement.

The implication for surgical planning is that attention should be paid not only to ventricular entry but also to valve site and tract routing. While our series was not designed to evaluate hardware types or positions, it seems plausible that more lateral or parietal placement, or avoidance of the nuchal line corridor, might reduce nerve irritation. Future strategies could include preoperative mapping of occipital nerve pathways to guide tunneling and valve pocket location, especially in patients with prior neuralgic pain syndromes.

Several limitations warrant consideration. First, the retrospective design precluded systematic application of ICHD-3 diagnostic criteria. We were unable to determine how many of our 24 patients would strictly fulfill all ICHD-3 requirements, as structured headache assessments and standardized neurological examinations were not performed prospectively. Physical examination findings such as focal tenderness were documented in most but not all cases, and we cannot exclude the possibility that some patients lacked findings that would be required for formal ICHD-3 diagnosis. Future prospective studies using standardized ICHD-3 assessment tools are needed to validate our operational criteria and determine their sensitivity and specificity relative to the international standard.

Second, we relied heavily on response to occipital nerve blocks as a diagnostic criterion. However, nerve blocks are known to have substantial placebo effects in headache disorders, with placebo response rates reported between 20–40% in various headache types. All blocks in our series were performed open-label without sham or placebo controls, and response assessment was based on subjective patient report rather than validated outcome measures. Therefore, we cannot definitively distinguish true diagnostic specificity from placebo-mediated or non-specific analgesic effects. Some patients classified as Occipital Neuralgia based on favorable block response may have had other pain mechanisms. Placebo-controlled or sham-controlled nerve block studies would be required to establish the true diagnostic value of this intervention in the post-shunt headache population.

Third, given the small number of affected patients (*n* = 24), statistical power for subgroup analyses and multivariable modeling is limited. Conclusions regarding risk factors, predictors of treatment response, and comparative effectiveness of different interventions must be considered exploratory and hypothesis-generating rather than definitive. Larger multicenter cohorts would be needed to identify clinical or radiological predictors of Occipital Neuralgia after shunt placement.

Finally, despite our efforts to exclude shunt malfunction through clinical assessment and neuroimaging, we cannot entirely rule out subtle shunt-related drainage abnormalities contributing to symptoms in some cases. Ventricular size on imaging is an imperfect marker of shunt function, particularly in long-standing shunts where ventricular compliance may be altered. Some patients classified as having isolated Occipital Neuralgia may have had concurrent minor shunt dysfunction that was not detected by available diagnostic modalities.

## Conclusions

Occipital Neuralgia is a recognizable syndrome of post-shunt headache in adults, characterized by occipital neuropathic pain attributable to irritation of the occipital nerves by shunt hardware. It should be suspected in patients with lancinating occipital pain and scalp tenderness after VP shunt placement, especially when imaging excludes typical shunt complications. In our series, Occipital Neuralgia accounted for most post-shunt headaches and responded far better to peripheral nerve interventions than to shunt revisions. Incorporating this entity into the differential diagnosis of post-shunt headache allows clinicians to tailor management appropriately—often with minimally invasive, nerve-targeted procedures—and avoid unwarranted shunt surgeries. We recommend a multidisciplinary approach to post-shunt headaches, partnering neurosurgeons with pain specialists to promptly identify Occipital Neuralgia and implement targeted therapy.

Further prospective studies are needed to refine diagnostic criteria, evaluate preventive measures (like shunt placement strategies), and confirm the long-term benefits of nerve-targeted treatments in this unique patient population, including prospective studies with standardized ICHD-3 assessments and placebo-controlled nerve block protocols to establish diagnostic specificity.

## Data Availability

The data supporting the findings of this study are presented within the article. Additional de-identified data may be made available from the corresponding author upon reasonable request and subject to institutional and ethics approvals, where applicable.
